# Genome-Wide Quantitative Trait Locus Mapping Identifies Multiple Major Loci for Brittle Rachis and Threshability in Tibetan Semi-Wild Wheat (*Triticum aestivum* ssp. *tibetanum* Shao)

**DOI:** 10.1371/journal.pone.0114066

**Published:** 2014-12-04

**Authors:** Yun-Feng Jiang, Xiu-Jin Lan, Wei Luo, Xing-Chen Kong, Peng-Fei Qi, Ji-Rui Wang, Yu-Ming Wei, Qian-Tao Jiang, Ya-Xi Liu, Yuan-Ying Peng, Guo-Yue Chen, Shou-Fen Dai, You-Liang Zheng

**Affiliations:** 1 Triticeae Research Institute, Sichuan Agricultural University, Chengdu, Sichuan, People’s Republic of China; 2 Key Laboratory of Southwestern Crop Germplasm Utilization, Ministry of Education, Sichuan Agricultural University, Chengdu, Sichuan, People’s Republic of China; Zhejiang University, China

## Abstract

Tibetan semi-wild wheat (*Triticum aestivum* ssp. *tibetanum* Shao) is a semi-wild hexaploid wheat resource that is only naturally distributed in the Qinghai-Tibet Plateau. Brittle rachis and hard threshing are two important characters of Tibetan semi-wild wheat. A whole-genome linkage map of *T. aestivum* ssp. *tibetanum* was constructed using a recombinant inbred line population (Q1028×ZM9023) with 186 lines, 564 diversity array technology markers, and 117 simple sequence repeat markers. Phenotypic data on brittle rachis and threshability, as two quantitative traits, were evaluated on the basis of the number of average spike rachis fragments per spike and percent threshability in 2012 and 2013, respectively. Quantitative trait locus (QTL) mapping performed using inclusive composite interval mapping analysis clearly identified four QTLs for brittle rachis and three QTLs for threshability. However, three loci on 2DS, 2DL, and 5AL showed pleiotropism for brittle rachis and threshability; they respectively explained 5.3%, 18.6%, and 18.6% of phenotypic variation for brittle rachis and 17.4%, 13.2%, and 35.2% of phenotypic variation for threshability. A locus on 3DS showed an independent effect on brittle rachis, which explained 38.7% of the phenotypic variation. The loci on 2DS and 3DS probably represented the effect of *Tg* and *Br1*, respectively. The locus on 5AL was in very close proximity to the *Q* gene, but was different from the predicted *q* in Tibetan semi-wild wheat. To our knowledge, the locus on 2DL has never been reported in common wheat but was prominent in *T. aestivum* ssp. *tibetanum* accession Q1028. It remarkably interacted with the locus on 5AL to affect brittle rachis. Several major loci for brittle rachis and threshability were identified in Tibetan semi-wild wheat, improving the understanding of these two characters and suggesting the occurrence of special evolution in Tibetan semi-wild wheat.

## Introduction

Cereal crops, the world’s primary food source, have been domesticated from a diverse array of grass species. In wheat, domestication has occurred at all three ploidy levels. The cultivated forms of wheat are *Triticum monococcum* ssp. *monococcum* (2n = 2x = 14) at the diploid level, *Triticum turgidum* ssp. *durum* (2n = 4x = 28) at the tetraploid level, and *Triticum aestivum* ssp. *aestivum* (2n = 6x = 42) at the hexaploid level. The major traits subjected to selection include loss of spike shattering, loss of tough glumes, increased seed size, reduced number of tillers, change in plant architecture, and reduced seed dormancy [1].

Unlike cultivated wheat, wild wheat shows characteristic traits such as brittle rachis, tough glume, and hard-threshing features, which help the wild species survive and multiply in nature. In polyploid wheats, a major modifier gene for a domestication-related trait (*q* gene) has been identified on the long arm of chromosome 5A; this gene affects brittle rachis, tough glume, and hard-threshing features [2]–[11]. *Q* gene is well known and has been cloned and is known to be a member of the APETALA2 family of transcription factors [8], [10]. The tenacious glume trait was found to be controlled by *Tg* gene, which was mapped on the short arm of group-2 chromosomes [12]–[16]. *Tg* also has a strong effect on threshability, which could be simultaneous detected by threshability and glume tenacity [12], [13]. The brittle rachis trait is primarily controlled by *Br* gene, which was mapped on homoeologous group 3 chromosomes [17]–[22] and chromosome 2A [23]. Unlike *Tg*, *Br* is not linked with threshability and showed an independent effect on brittle rachis [23].

Tibetan semi-wild wheat (*T. aestivum* ssp. *tibetanum* Shao) is an endemic hexaploid wheat group in China; it is found only in the Qinghai-Tibet Plateau of China and was recognized as the subspecies of the hexaploid wheat [24]. The typical primitive and classified characters of Tibetan semi-wild wheat are spontaneous spike disarticulation and tough glume, indicating that it is closer to the wild wheat than other existing *T. aestivum* species [24]. Spike disarticulation pattern of the Tibetan semi-wild wheat is wedge type, and its disarticulation occurs above the junction of the rachis with the rachilla, unlike that in spelt wheat, which occurs below the junction of the rachis and rachilla [25]. In contrast to spelt wheat, Tibetan semi-wild wheat rachis are fractured much more easily [26].

The brittle rachis of Tibetan semi-wild wheat was mapped to the short arm of chromosome 3D and designated as *Br1* [18], [26], [27]. However, *Br1* might be not the only gene responsible for brittle rachis in Tibetan semi-wild wheat. Cao et al. [25] suggested that three brittle rachis genes were involved in the progenies of hybrid of Tibetan semi-wild wheat with spelt wheat. Chen et al. [28] supported the result and believed that other genes for brittle rachis might exist. In our study, many intermediate types of brittle rachis were found in the population from Q1028×ZM9023, suggesting that several genes for brittle rachis might exist in Q1028. Previously, the lack of genome-wide molecular marker analysis might have hindered the identification of potential genes for brittle rachis in Tibetan semi-wild wheat. Moreover, further studies are needed to explain the reason for extremely brittle rachis in Tibetan semi-wild wheat and to determine whether this trait is controlled by a single gene or multiple major genes. Investigation of the control of domestication-related traits such as brittle rachis and threshability might improve our knowledge on the evolution of Tibetan semi-wild wheat.

To better understand the genetic control of brittle rachis and threshability in Tibetan semi-wild wheat, we constructed a high-density genetic map by using a recombinant inbred line (RIL) population and multiple types of molecular markers, including diversity array technology (DArT) and simple sequence repeats (SSRs). This quantitative trait locus (QTL) mapping revealed new QTLs for brittle rachis and threshability in *T. aestivum* ssp. *tibetanum*.

## Materials and Methods

### Plant materials

The 186 RILs used in the present study were developed using a single-seed-descent method from a cross between Tibetan semi-wild wheat accessions Q1028 (brittle rachis; hard threshing) and ZM9023 (tough rachis; free threshing). In October 2011 and 2012 respectively, 186 RILs at generations F_10_ and F_11_ and the parents of these RILs were planted in the experimental field of Triticeae Research Institute, Sichuan Agricultural University, China. Each line was single-seed planted in two 2-m long rows, with 30-cm distance between rows and 10-cm spacing within rows. Field management followed common practices for wheat production.

### Assessment of brittle rachis and threshability

In May 2012 and 2013, after harvest, the 186 RILs were kept under indoor ventilation and air-dried for about one week to ensure that all the spikes had relatively identical moisture content.

In this study, brittle rachis and threshability were evaluated as two quantitative traits [12]. Five randomly chosen spikes of each line were passed through an electric-powered single-plant thresher (2800 r/min), and the number of average spike rachis fragments (RF) were measured after mechanical threshing. Threshability (free threshing, FT) was calculated as the percentage of completely threshed seeds from all seeds harvested.

### Molecular marker analyses

We used SSR and DArT markers to construct a high-density genetic map of Tibetan semi-wild wheat. Genomic DNA was extracted from fresh leaf tissue (1-week-old seedlings) by using the cetyltrimethylammonium bromide method [29]. A total of 1,089 SSR primers were tested for polymorphisms, and 118 polymorphic SSR markers between the parents were used to screen the RIL populations. For SSR analysis, polymerase chain reaction (PCR) was performed as described by Sood et al. [14]. After the PCR products were electrophoresed on 8% polyacrylamide gels, amplified fragments were detected by silver staining.

DArT was used to identify polymorphic markers for the construction of a linkage map. DNA from the 186 lines and two parents were sent to Diversity Arrays Technology Pvt. Ltd. (http://www.diversityarrays.com) for whole-genome profiling. The procedures used for hybridization of DNA to the DArT array and for image analysis and polymorphism scoring were according to those described by Wenzl et al. [30].

### Data analysis and QTL mapping

SPSS version 17.0 for Windows (SPSS Inc., Chicago, IL) was used for basic statistical analyses and graph production. JointMap 4 [31] was used for linkage analysis of marker data. A minimum limit of detection (LOD) of ≧3.0 was used to develop the linkage map. Recombination frequencies were converted to centimorgans (cM) by using the Kosambi mapping function. The segregation ratio of each marker and its deviation from the expected ratio were evaluated using the chi-square test. QTL analysis was conducted using QTL IciMapping version 3.2 [32] following inclusive composite interval mapping (ICIM) [33]. An LOD score of ≧2.5 was used to detect a QTL, and the walk speed was 1.0 cM. The QTLNetwork program version 2.1 based on a mixed linear model [34] was used to identify the epistatic QTL for brittle rachis and threshability in joint analysis of the phenotypic values for the data collected over two years. Briefly, the values for testing window and filtration window were set at 10 cM, and the walking speed was 1 cM. The LOD threshold of QTL was determined using a 1,000 permutation test at 95% confidence level. The proportion of observed phenotypic variance explained by each additive and epistatic QTL and the corresponding additive effects were also estimated.

## Results

### Analysis of phenotypic data

**Figure 1:**
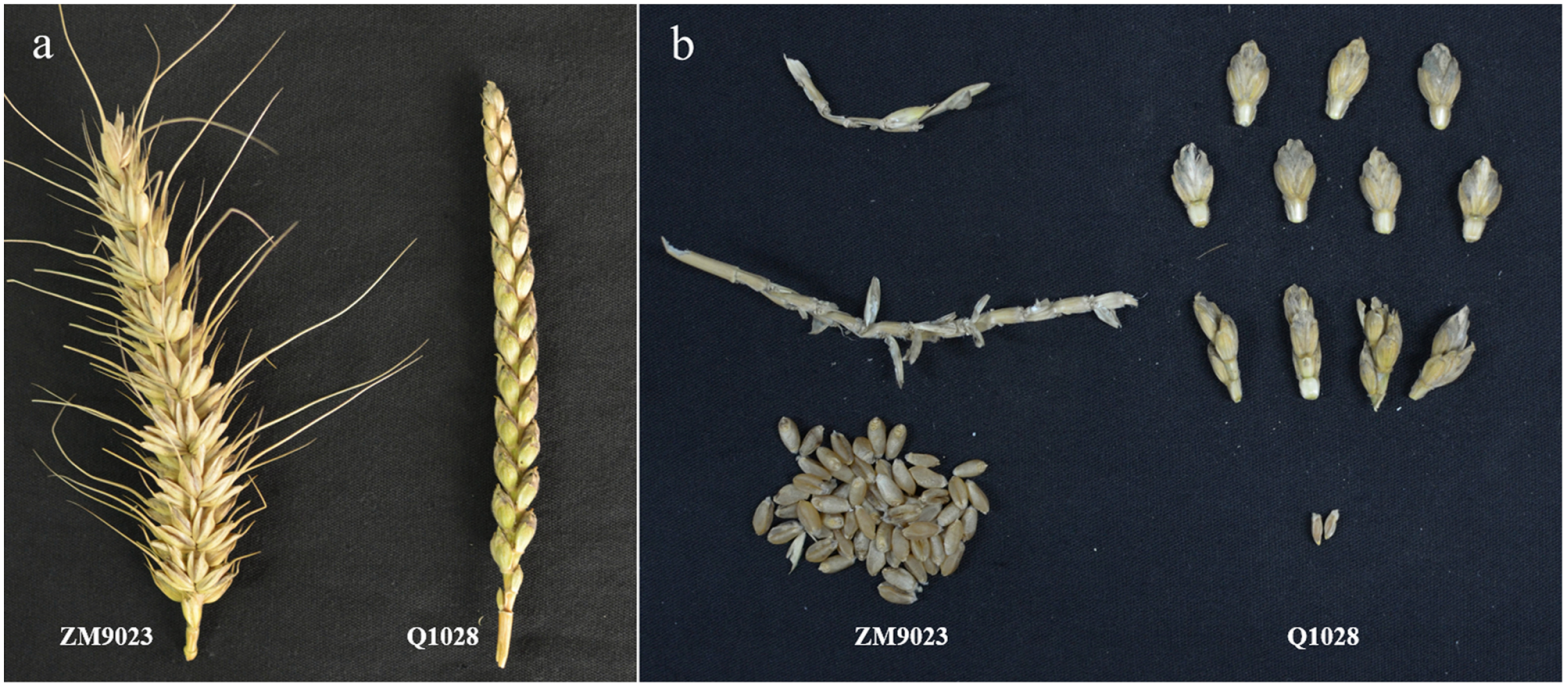
Spikes and phenotypes after mechanical threshing. **a** Comparison of spikes between ZM9023 and *Triticum aestivum* ssp. *tibetanum* Shao accession Q1028. **b** The phenotypes after mechanical threshing between ZM9023 and *Triticum aestivum* ssp. *tibetanum* Shao accession Q1028 showed significant difference both in brittle rachis and threshability.

**Figure 2:**
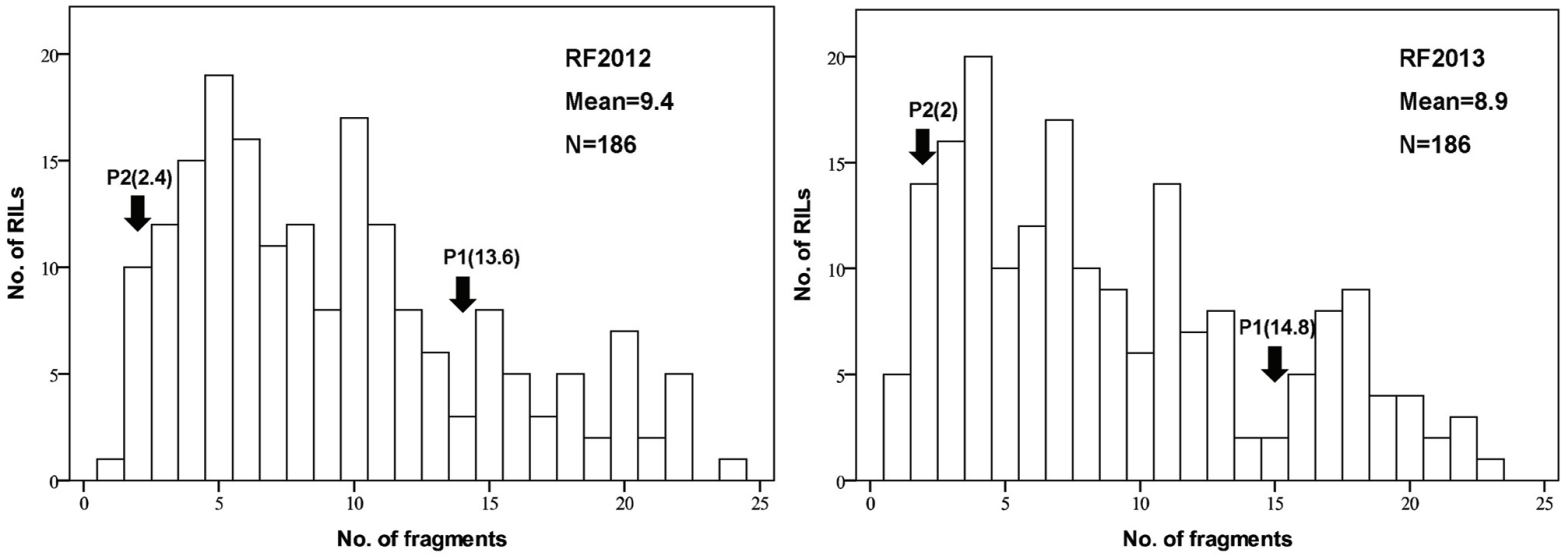
Frequency distributions of the number of average spike rachis fragments. RF2012 and RF2013 were obtained from crosses between *Triticum aestivum* ssp. *tibetanum* Shao population Q1028×ZM9023 established in 2012 and 2013, respectively. P1: Q1028, P2: ZM9023. RILs: recombinant inbred lines. Mean: average value of the trait. N: the number of RILs.

**Figure 3:**
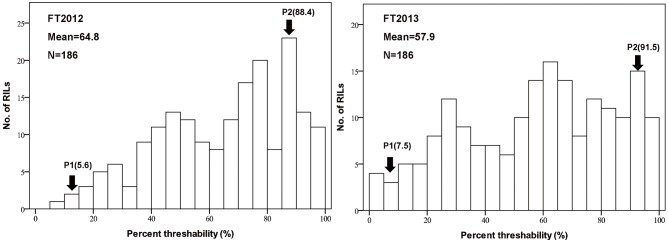
Frequency distributions of the percentage of completely threshed seeds from all seeds harvested. FT2012 and FT2013 were obtained from crosses between *Triticum aestivum* ssp. *tibetanum* Shao population Q1028×ZM9023 established in 2012 and 2013, respectively. P1: Q1028, P2: ZM9023. RILs: recombinant inbred lines. Mean: average value of the trait. N: the number of RILs.

The two parents, Q1028 and ZM9023, showed significant differences in both brittle rachis and threshability (Figs. 1–3). The brittle rachis of Q1028 was 13.6–14.8, which was significantly higher than that of ZM9023 (2–2.4), whereas the threshability of Q1028 was 5.6%–7.5%, which was significantly lower than that of ZM9023 (88.42%–91.50%). The mean values of brittle rachis and threshability in the RIL populations in 2012 and 2013 were 8.94–9.36 and 57.85%–64.78%, respectively (Table 1). All data for the two trials for two years showed continuous distribution in the RIL populations (Figs. 2 and 3). The correlation coefficients (*R*) of the trials between the two years were 0.93 for brittle rachis and 0.94 for threshability, indicating that the two traits remained relatively stable (Table 2). There were significant and negative correlations between brittle rachis and threshability, with *R* ranging from −0.49 to −0.52 (Table 2).

**Table 1:** Phenotypic values for brittle rachis and threshability in *Triticum aestivum* ssp. *tibetanum* Shao population from accessions Q1028×ZM9023.

Trait type	Trait^a^	Parent		Population (186 recombinant inbred lines)			
		Q1028	ZM9023	Minimum	Maximum	Mean	Standard error
Brittle rachis	RF2012	13.6	2.4	1	24	9.4	5.4
	RF2013	14.8	2	1	23	9.0	5.7
Threshability (%)	FT2012	5.6	88.4	5.7	99.6	64.8	22.6
	FT2013	7.5	91.5	1.4	100	57.9	26.4

a Brittle rachis is expressed as the number of average spike rachis fragments per spike generated after threshing; the two trials conducted were designated as RF2012 and RF2013. Threshability is expressed as the percentage of threshed seeds (the number of threshed seeds/total seeds×100); the two trials conducted were designated as FT2012 and FT2013.

**Table 2:** Correlation coefficients of brittle rachis and threshability in *Triticum aestivum* ssp. *tibetanum* Shao population from accessions Q1028×ZM9023.

Trial^a^	RF2012	RF2013	FT2012	FT2013
RF2012	1			
RF2013	0.94**	1		
FT2012	−0.51**	−0.52**	1	
FT2013	−0.49**	−0.50**	0.93**	1

**Indicates significant levels at *p*<0.01.

a For brittle rachis, the two trials conducted were designated as RF2012 and RF2013. For threshability, the two trials conducted were designated as FT2012 and FT2013.

### Construction of the genetic map

A whole-genome genetic map was constructed using 186 RIL populations by using 564 DArT and 117 SSR markers, which were distributed in 22 linkage groups and covered a genetic distance of 2727 cM (Figure S1). The distribution of markers was not uniform across the genome. Coverage of most chromosomes was obtained except for 4D and 5D.

### The major QTLs for brittle rachis

**Figure 4:**
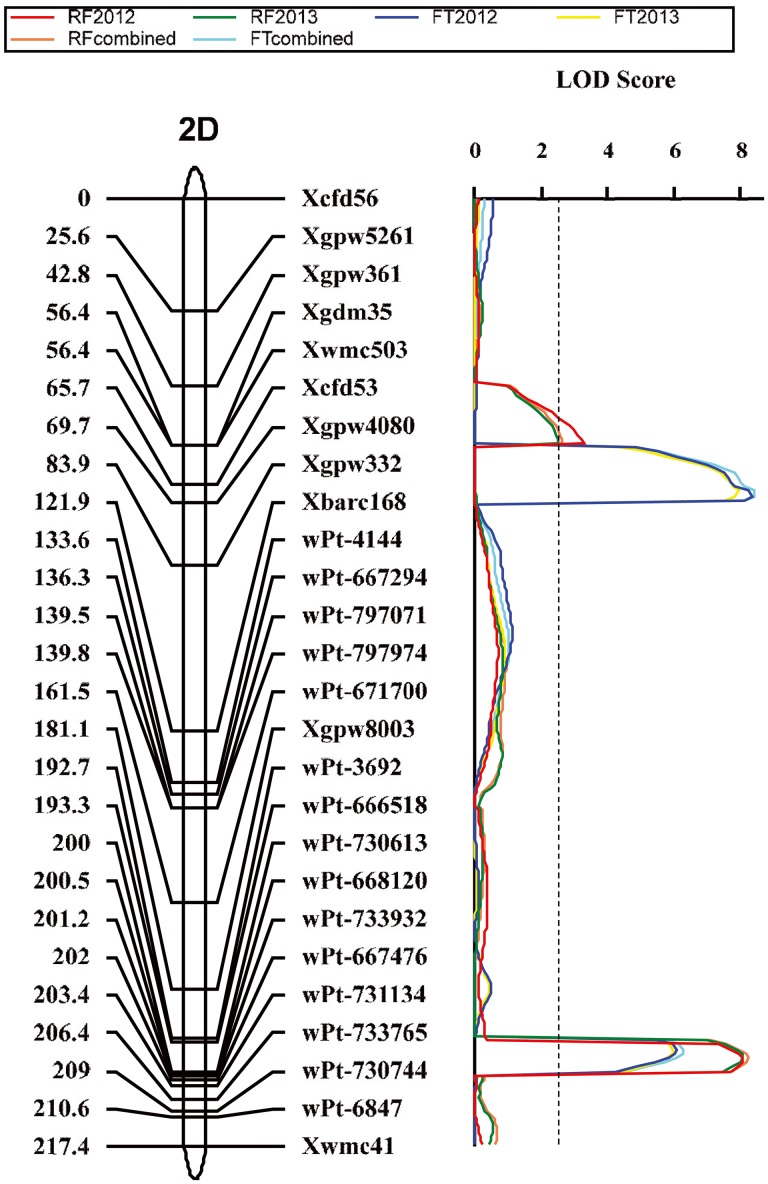
Quantitative trait loci (QTLs) for brittle rachis and threshability. QTL detected on chromosome 2D in the *Triticum aestivum* ssp. *tibetanum* Shao population from accessions Q1028×ZM9023. Marker positions are shown on the right of the linkage map, and distances between loci (in centimorgans, cM) are shown on the left. The vertical dashed line indicates the significance threshold (LOD = 2.5).

**Figure 5:**
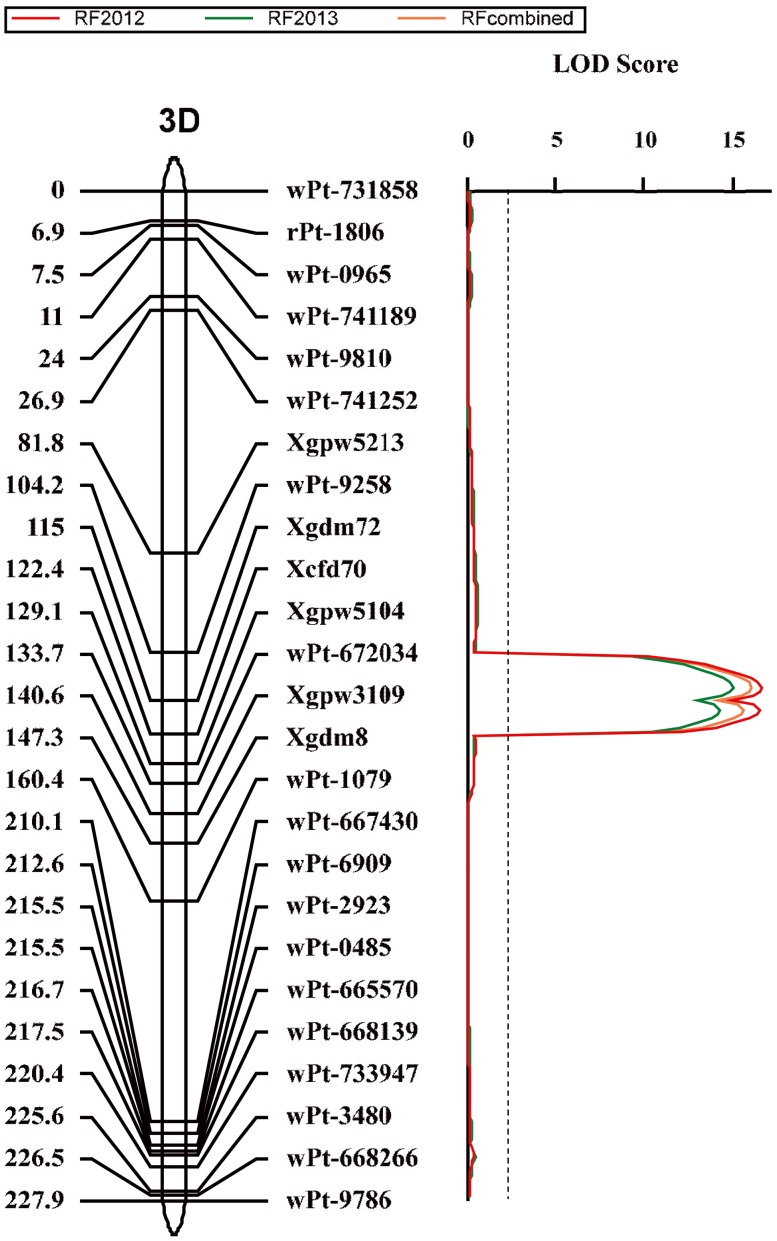
Quantitative trait loci (QTL) for brittle rachis. QTL detected on chromosome 3D in the *Triticum aestivum* ssp. *tibetanum* Shao population from accessions Q1028×ZM9023. Marker positions are shown on the right of the linkage map, and distances between loci (in centimorgans, cM) are shown on the left. The vertical dashed line indicates the significance threshold (LOD = 2.5).

**Figure 6:**
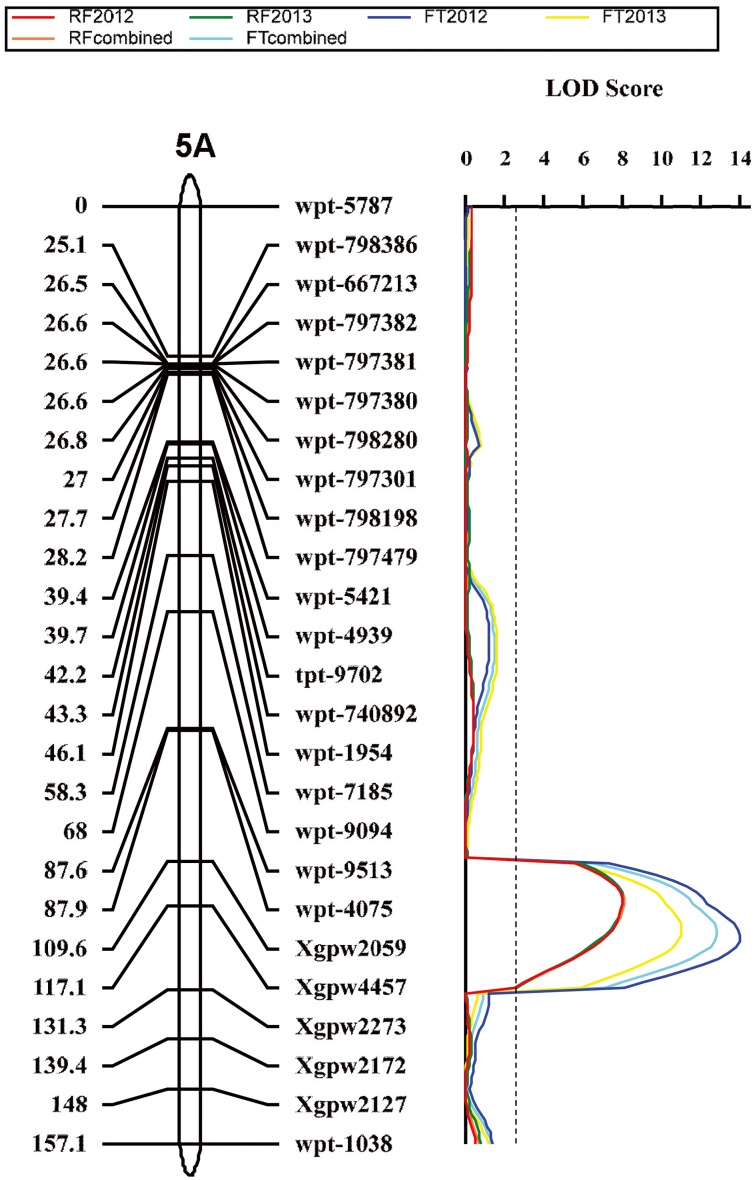
Quantitative trait loci (QTL) for brittle rachis and threshability. QTL detected on chromosome 5A in the *Triticum aestivum* ssp. *tibetanum* Shao population from accessions Q1028×ZM9023. Marker positions are shown on the right of the linkage map, and distances between loci (in centimorgans, cM) are shown on the left. The vertical dashed line indicates the significance threshold (LOD = 2.5).

Four QTLs for brittle rachis were identified in the mapping populations by using ICIM analysis, and all the positive alleles were derived from the brittle rachis parent, Q1028 (Table 3). All QTLs were significantly detected in the two years (Table 3). The range of phenotypic variation explained (PVE) was 5.3%–38.7% for all QTLs in the general average data of brittle rachis, and the total PVE of brittle rachis was >79%. The QTLs of brittle rachis were detected on chromosome arms 2DS, 2DL, 3DL, and 5AL (Figs. 4–6), which were designated *Qbr.sau-2D1, Qbr.sau-2D2*, *Qbr.sau-3A*, and *Qbr.sau-5A*, respectively, following the convention that “br” and “sau” stand for “brittle rachis” and “Sichuan Agricultural University,” respectively.

**Table 3:** Quantitative trait loci (QTLs) for threshability and brittle rachis identified in the *Triticum aestivum* ssp. *tibetanum* Shao population from accessions Q1028×ZM9023 by using the inclusive composite interval mapping (ICIM) analysis.

Trial type	QTL	Chromosome^a^	Trial	Position^b^	Left Marker	Right Marker	LOD	PVE (%)	Additive effect
Brittle Rachis	*Qbr.sau-2D1*	2DS	RF2012	56	Xgpw361	Xgdm35	3.3	6.6	1.4
			RF2013	56	Xgpw361	Xgdm35	2.5	5.1	1.3
			RF combined	56	Xgpw361	Xgdm35	2.6	5.3	1.3
	*Qbr.sau-2D2*	2DL	RF2012	197	wPt-666518	wPt-730613	8.1	18.2	2.4
			RF2013	197	wPt-666518	wPt-730613	8.1	18.4	2.5
			RF combined	197	wPt-666518	wPt-730613	8.2	18.6	2.4
	*Qbr.sau-3D*	3DS	RF2012	112	wPt-9258	Xgdm72	16.6	39.3	3.5
			RF2013	112	wPt-9258	Xgdm72	15.0	36.9	3.5
			RF combined	112	wPt-9258	Xgdm72	16.1	38.7	3.5
	*Qbr.sau-5A*	5AL	RF2012	117	Xgpw2059	Xgpw4457	7.4	16.7	2.2
			RF2013	116	Xgpw2059	Xgpw4457	8.1	18.9	2.5
			RF combined	116	Xgpw2059	Xgpw4457	7.9	18.6	2.4
Threshability	*Qft.sau-2D1*	2DS	FT2012	68	Xcfd53	Xgpw4080	8.3	17.5	−9.5
			FT2013	67	Xcfd53	Xgpw4080	7.9	16.2	−10.7
			FT combined	68	Xcfd53	Xgpw4080	8.4	17.4	−10.1
	*Qft.sau-2D2*	2DL	FT2012	195	wPt-666518	wPt-730613	6.1	13.1	−8.3
			FT2013	195	wPt-666518	wPt-730613	5.9	12.5	−9.5
			FT combined	195	wPt-666518	wPt-730613	6.3	13.2	−8.9
	*Qft.sau-5A*	5AL	FT2012	121	Xgpw4457	Xgpw2273	14.1	38.3	−14.2
			FT2013	122	Xgpw4457	Xgpw2273	11.1	29.9	−14.5
			FT combined	122	Xgpw4457	Xgpw2273	12.9	35.2	−14.4

LOD: limit of detection; PVE: phenotypic variation explained.

a Markers on the chromosome arm are considered to be linked with the QTL.

b The position of LOD score peak in the chromosomes (Figs. 4–6).

*Qbr.sau-2D1*, which was located on the 2D short arm region between Xgpw361 and Xgdm35 (Fig. 4), showed a minor effect on brittle rachis and explained 6.6% and 5.1% of phenotypic variation in 2012 and 2013, respectively (Table 3).

*Qbr.sau-2D2*, which was located on the 2D long arm region between wPt-666518 and wPt-730613, was considerably distant from *Qbr.sau-2D1* (Fig. 4). It was a major QTL for brittle rachis and explained 18.2% and 18.4% of phenotypic variation in 2012 and 2013, respectively (Table 3).

*Qbr.sau-3D*, the most significant QTL for brittle rachis, was located on the 3DS region between wPt-9258 and wPt-672034 (Fig. 5) and explained 39.3% and 36.9% of phenotypic variation in 2012 and 2013, respectively.

*Qbr.sau-5A*, which was located on 5A long arm region between Xgpw2059 and Xgpw4457 (Fig. 6) explained 16.6% and 17.0% of phenotypic variation in 2012 and 2013, respectively.

### The major QTLs for threshability

A total of three QTLs for threshability were identified by the ICIM analysis, and all the negative alleles were derived from the hard-threshing parent, Q1028 (Table 3). All QTLs were significantly detected in the two years (Table 3). The range of PVE was 13.2%–27.7% for all QTLs in the FT combined, and the total PVE was >65%. QTLs were detected on chromosome arms 2DS, 2DL, and 5AL (Figs. 4 and 6), which were designated *Qft.sau-2D1*, *Qft.sau-2D2*, and *Qft.sau-5A*, respectively, following the convention that “ft” and “sau” stand for “free threshing” and “Sichuan Agricultural University,” respectively. Moreover, the three QTLs for brittle rachis and threshability on 2DS, 2DL, and 5AL were located in very close proximity.

*Qft.sau-2D1* was located on 2D short arm region between Xcfd53 and Xgpw4080 (Fig. 4), and a QTL (*Qbr.sau-2D1*) with a minor effect on brittle rachis was found in the adjacent region. *Qft.sau-2D1* was a major QTL for threshability. It explained 17.5% and 16.2% of phenotypic variation in 2012 and 2013, respectively.

*Qft.sau-2D2*, which was located on the same region as *Qbr.sau-2D2* between wPt-3692 and wPt-7160 (Fig. 4), explained 13.1% and 12.5% of phenotypic variation in 2012 and 2013, respectively. Its effect on threshability was slightly lower than that of *Qft.sau-2D1.*

*Qft.sau-5A*, which was located on the same region as *Qbr.sau-5A* between Xgpw4457 and Xgpw2273 (Fig. 6), accounted for 38.3% and 29.9% of the phenotypic variation in 2012 and 2013, respectively.

### Epistatic analysis for brittle rachis and threshability

**Figure 7:**
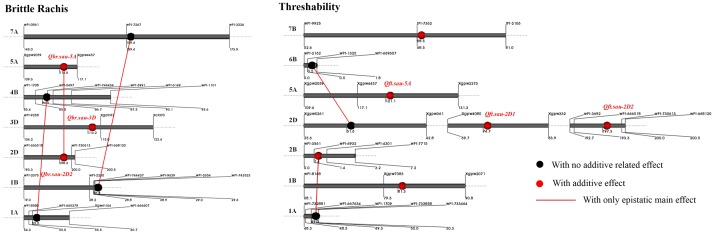
Location of additive and epistatic quantitative trait loci (QTLs) for brittle rachis and threshability on linkage groups in joint analysis of the two-year data. The chromosome names are listed on the left of the *bar*, the map distances (cM) are shown below the *bar*, and the markers are shown above the *bar*. The major additive loci are annotated in red color.

Three and two epistases were detected for brittle rachis and threshability, respectively (Fig. 7 and Table 4). All of them showed only epistatic main effect and non-significant epistatic × environment interaction. Further, two major additive QTLs (*Qbr.sau-2D2* and *Qft.sau-5A*) showed a significant interaction in brittle rachis. Three minor additive QTLs for threshability could be detected using QTLNetwork 2.1 (Table S1).

**Table 4:** Epistases for brittle rachis and threshability in the *Triticum aestivum* ssp. *tibetanum* Shao population from accessions Q1028×ZM9023.

Trait type	Loci(i)^a^			Loci(j)^a^			A^b^	PVE (%)
	Chr.	Left	Right	Chr.	Left	Right		
Brittle Rachis	2D	wPt-666518	wPt-730613	5A	Xgpw2059	Xgpw4457	0.60**	1.5
	1A	Xgwm164	wPt-666607	4B	wPt-5497	wPt-744434	0.96**	3.4
	1B	wPt-744437	wPt-9639	7A	wPt-7267	wPt-3226	0.97**	2.8
Threshability	1A	wPt-1709	wPt-733858	2B	wPt-6932	wPt-4301	3.13**	1.5
	2D	Xgpw5261	Xgpw361	6B	wPt-1325	wPt-669607	−6.52**	4.6

**Indicates significant levels at *p*<0.01.

a Markers on the chromosome arm are considered to be linked with the QTL.

b A represents the estimated additive effect of epistatic QTL.

## Discussion

### Multiple major loci for brittle rachis and threshability in Tibetan semi-wild wheat

The effect of several major genes for brittle rachis and threshability were identified in Tibetan semi-wild wheat by using the ICIM analysis. Four QTLs for brittle rachis were identified on 2DS, 2DL, 3DS, and 5AL; three QTLs for threshability were identified on 2DS, 2DL, and 5AL. However, three loci on 2DS, 2DL, and 5A simultaneously controlled brittle rachis and threshability, which could explain the significant correlation between brittle rachis and threshability.

Two loci for brittle rachis and threshability on 2D were identified in the RILs in this study. The reported major gene for threshability in 2D was *Tg*, which was reported to be located on 2D short arm. *Qbr.sau-2D1* and *Qft.sau-2D1* were located in an adjacent region on chromosome 2D short arm, which was very close to the region containing *Tg* [13], [14]. Previous reports showed that *Tg* was the major gene for glume tenacity on 2DS in Tibetan semi-wild wheat [25], [35]. Therefore, the effect of *Qbr.sau-2D1* should be derived from *Tg,* which was also reported to affect brittle rachis and threshability [12], [13].

Another locus was located on the 2D long arm region between wPt-666518 and wPt-730613 with a 6.7-cM flanking interval. It explained 18.6% and 13.2% of phenotypic variation in brittle rachis and threshability, respectively, and had a major effect on both brittle rachis and threshability. It interacted with the locus on 5AL to affect brittle rachis, probably via the regulatory relationships between them. This locus has never or rarely been reported in common wheat but was prominent in *T. aestivum* ssp. *tibetanum* accession Q1028. Therefore, it might be a special variation in Tibetan semi-wild wheat to allow its adaptation to the severe semi-wild environment in the Qinghai-Tibet Plateau.

A major locus for brittle rachis and threshability was identified on 5AL of Tibetan semi-wild wheat, which was not reported previously [7]. Comparisons of our genetic map with those reported previously revealed that this QTL was located very close to the *Q* gene. However, the locus in Tibetan semi-wild wheat was speculated to be different from the well-known *q* gene in *Triticum spelta* and was almost the same as that found in the wheat line ‘Chinese Spring’, i.e., domestication type, by sequencing (unpublished data). Further studies are warranted to perform detailed analysis on this locus.

A QTL for brittle rachis was identified on 3DS as expected. Several previous studies indicated that the brittle rachis of Tibetan semi-wild wheat was governed by *Br1* [25], [26], which was located on the short arm of chromosome 3DS. In this study, *Qbr.sau-3D* was found to be a major QTL for brittle rachis, which explained 39.36% of phenotypic variation. It was located clearly in the chromosome 3DS region with a 10.8-cM interval between wPt-9258 and Xgdm72, which almost corresponded to the location of *Br1* locus reported by Watanabe et al. [18]. *Qbr.sau-3D* should be *Br1,* which was the most important locus for brittle rachis in Tibetan semi-wild wheat.

### Phylogenetics of Tibetan semi-wild wheat

Several major genes for brittle rachis and threshability were identified in Tibetan semi-wild wheat; the identification of these genes not only improved the understanding of brittle rachis and threshability in wheat but also revealed a possibility of special evolution in Tibetan semi-wild wheat. The brittle rachis feature in Tibetan semi-wild was significantly different from that in spelt wheat [25]. The brittle rachis of spelt wheat was controlled by the *q* gene, which showed pleiotropism for brittle rachis, tough glume, and threshability characters. However, in Tibetan semi-wild wheat, brittle rachis was controlled by four genes. This suggested that there were entirely different systems for controlling brittle rachis between Tibetan semi-wild wheat and spelt wheat.

Hexaploid wheat has been speculated to have originated by hybridization of domesticated emmer with *Aegilops tauschii*. Tibetan semi-wild wheat was a subspecies of *T. aestivum* [24], which is a characteristic type of hexaploid wheat in China. The hexaploid ancestor of this wheat was thought to have originated from Middle East, rather than China [36]–[38]. However, although synthesized hexaploid wheat had the primitive D-genome from *Ae. tauschii* with a brittle rachis gene [17], it shows tough rachis character. The two major QTLs for brittle rachis located on 2DL and 3DS, which were identified in our study, have never been reported in any other hexaploid wheat, except that a gene for brittle rachis on *Br1* region was reported in a *Triticum aestivum-Aegilops tauschii* introgression line [19]. Our results implied that Tibetan semi-wild wheat could have originated by de-domestication, or an extra hybridization with *Ae. tauschii* relative to its original type. However, the characters of brittle rachis, tough glume, hard threshing, and strong seed dormancy [39] in Tibetan semi-wild wheat might have resulted from natural selection in the wild state.

## Supporting Information

Figure S1 **The linkage map of Q1028×ZM9023 by using 564 DArT and 117 SSR makers.** Numbers on the left are genetic distances in centiMorgan.
(PDF)

Table S1 **The additive QTLs for brittle rachis and threshability in joint analysis of two years data by QTLNetwork 2.1.**
(DOCX)
