# Supplementary material for: Genome-Wide Quantitative Trait Locus Mapping Identifies Multiple Major Loci for Brittle Rachis and Threshability in Tibetan Semi-Wild Wheat (Triticum aestivum ssp. tibetanum Shao)
Source: PLoS One. 2014 Dec 4;9(12):e114066. doi: 10.1371/journal.pone.0114066 (PMC4256410; doi:10.1371/journal.pone.0114066)
Supplement: Figure S1 — The linkage map of Q1028×ZM9023 by using 564 DArT and 117 SSR makers. Numbers on the left are genetic distances in centiMorgan. (PDF) [file pone.0114066.s001.pdf]

The linkage map of Q1028 × ZM9023

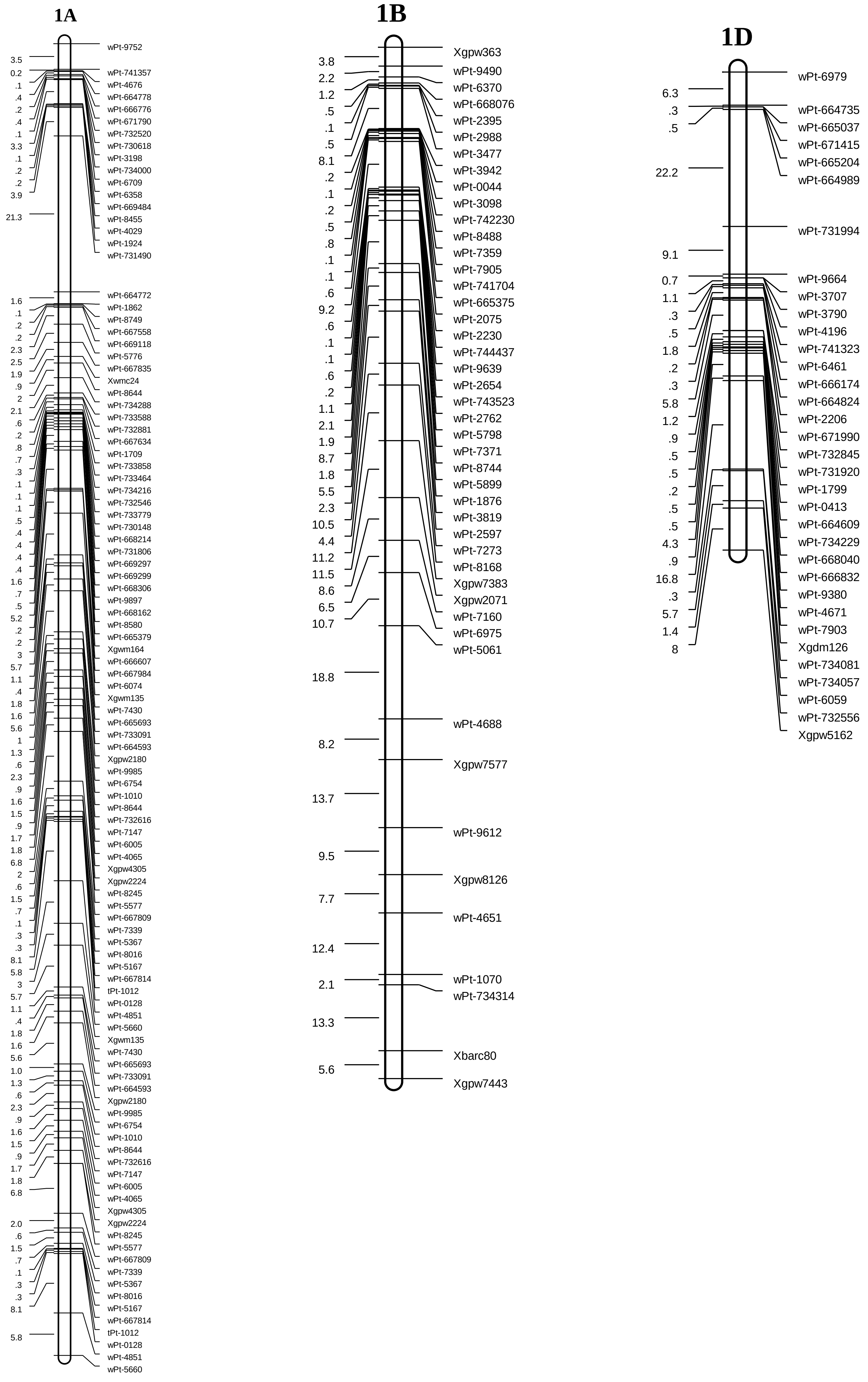

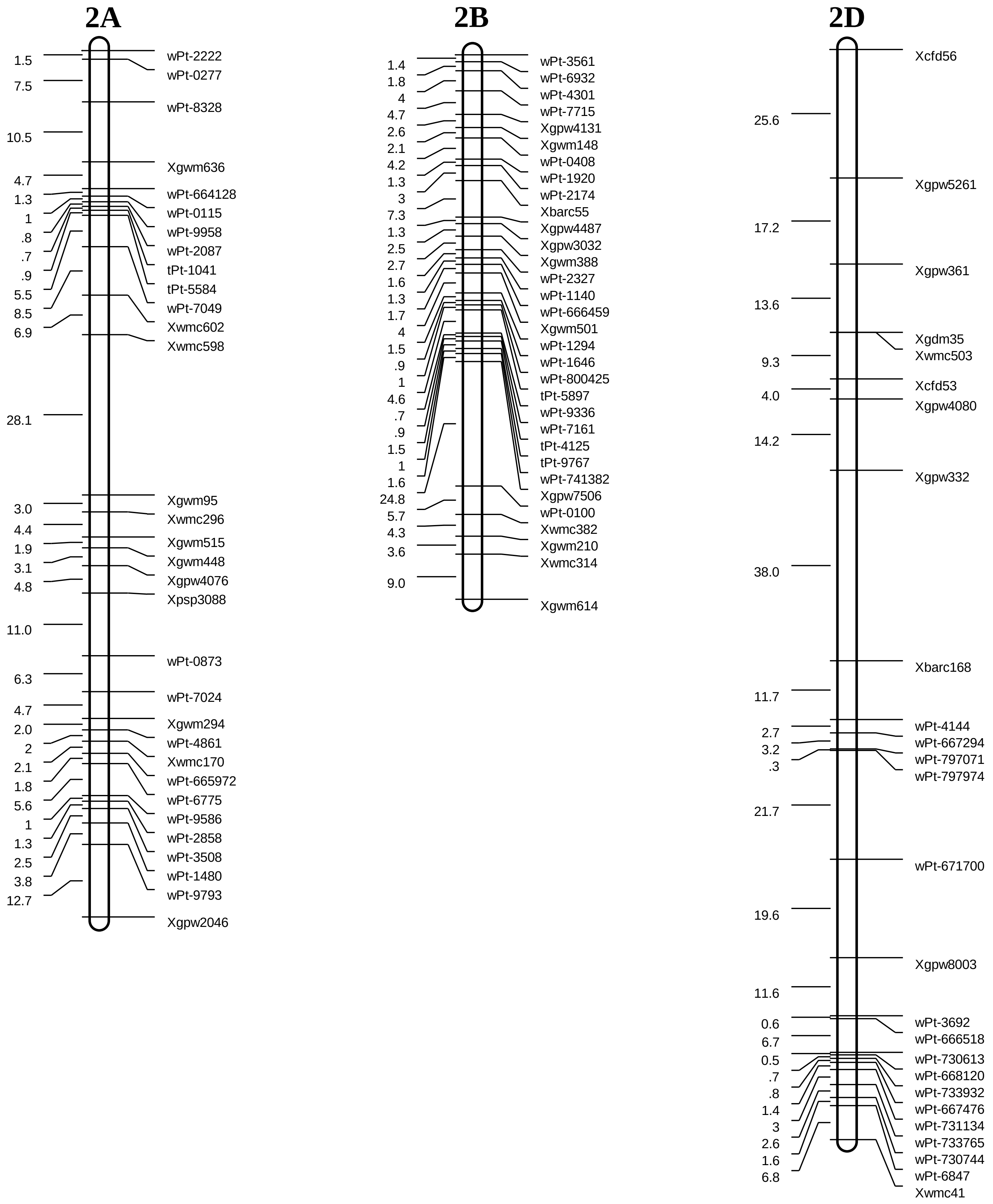
2A
2B
2D
wPt-2222
wPt-0277
wPt-8328
Xgwm636
wPt-664128
wPt-0115
wPt-9958
wPt-2087
tPt-1041
tPt-5584
wPt-7049
Xwmc602
Xwmc598
Xgwm95
Xwmc296
Xgwm515
Xgwm448
Xgpw4076
Xpsp3088
wPt-0873
wPt-7024
Xgwm294
wPt-4861
Xwmc170
wPt-665972
wPt-6775
wPt-9586
wPt-2858
wPt-3508
wPt-1480
wPt-9793
Xgpw2046
wPt-3561
wPt-6932
wPt-4301
wPt-7715
Xgpw4131
Xgwm148
wPt-0408
wPt-1920
wPt-2174
Xbarc55
Xgpw4487
Xgpw3032
Xgwm388
wPt-2327
wPt-1140
wPt-666459
Xgwm501
wPt-1294
wPt-1646
wPt-800425
tPt-5897
wPt-9336
wPt-7161
tPt-4125
tPt-9767
wPt-741382
Xgpw7506
wPt-0100
Xwmc382
Xgwm210
Xwmc314
Xgwm614
Xcfd56
Xgpw5261
Xgpw361
Xgdm35
Xwmc503
Xcfd53
Xgpw4080
Xgpw332
Xbarc168
wPt-4144
wPt-667294
wPt-797071
wPt-797974
wPt-671700
Xgpw8003
wPt-3692
wPt-666518
wPt-730613
wPt-668120
wPt-733932
wPt-667476
wPt-731134
wPt-733765
wPt-730744
wPt-6847
Xwmc41

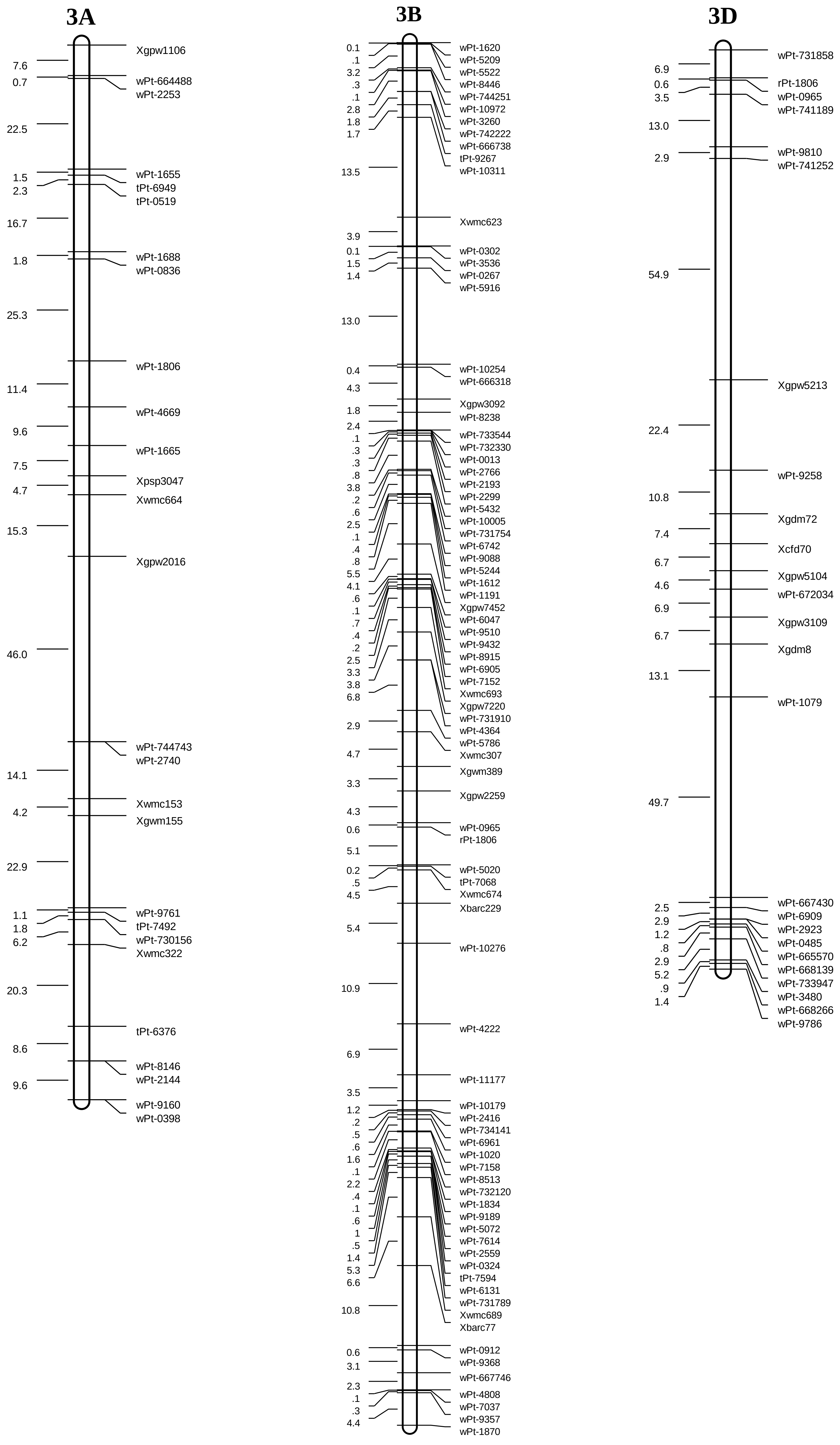
3A
Xgpw1106
7.6
0.7
wPt-664488
wPt-2253
22.5
1.5
2.3
wPt-1655
tPt-6949
tPt-0519
16.7
1.8
wPt-1688
wPt-0836
25.3
wPt-1806
11.4
wPt-4669
9.6
wPt-1665
7.5
Xpsp3047
4.7
Xwmc664
15.3
Xgpw2016
46.0
wPt-744743
wPt-2740
14.1
Xwmc153
4.2
Xgwm155
22.9
1.1
1.8
6.2
wPt-9761
tPt-7492
wPt-730156
Xwmc322
20.3
tPt-6376
8.6
wPt-8146
wPt-2144
9.6
wPt-9160
wPt-0398
3B
0.1
.1
3.2
.3
.1
2.8
1.8
1.7
wPt-1620
wPt-5209
wPt-5522
wPt-8446
wPt-744251
wPt-10972
wPt-3260
wPt-742222
wPt-666738
tPt-9267
wPt-10311
13.5
Xwmc623
3.9
0.1
1.5
1.4
wPt-0302
wPt-3536
wPt-0267
wPt-5916
13.0
0.4
4.3
wPt-10254
wPt-666318
1.8
Xgpw3092
wPt-8238
2.4
.1
.3
.3
.8
3.8
.2
.6
2.5
.1
.4
.8
5.5
4.1
.6
.1
.7
.4
.2
2.5
3.3
3.8
6.8
wPt-733544
wPt-732330
wPt-0013
wPt-2766
wPt-2193
wPt-2299
wPt-5432
wPt-10005
wPt-731754
wPt-6742
wPt-9088
wPt-5244
wPt-1612
wPt-1191
Xgpw7452
wPt-6047
wPt-9510
wPt-9432
wPt-8915
wPt-6905
wPt-7152
Xwmc693
Xgpw7220
wPt-731910
wPt-4364
wPt-5786
Xwmc307
2.9
4.7
Xgwm389
3.3
Xgpw2259
4.3
0.6
wPt-0965
rPt-1806
5.1
0.2
.5
4.5
wPt-5020
tPt-7068
Xwmc674
Xbarc229
5.4
wPt-10276
10.9
wPt-4222
6.9
wPt-11177
3.5
1.2
.2
.5
.6
1.6
.1
2.2
.4
.1
.6
1
.5
1.4
5.3
6.6
wPt-10179
wPt-2416
wPt-734141
wPt-6961
wPt-1020
wPt-7158
wPt-8513
wPt-732120
wPt-1834
wPt-9189
wPt-5072
wPt-7614
wPt-2559
wPt-0324
tPt-7594
wPt-6131
wPt-731789
Xwmc689
Xbarc77
10.8
0.6
3.1
wPt-0912
wPt-9368
wPt-667746
2.3
.1
.3
4.4
wPt-4808
wPt-7037
wPt-9357
wPt-1870
3D
wPt-731858
6.9
0.6
3.5
rPt-1806
wPt-0965
wPt-741189
13.0
2.9
wPt-9810
wPt-741252
54.9
Xgpw5213
22.4
wPt-9258
10.8
Xgdm72
7.4
Xcfd70
6.7
Xgpw5104
4.6
wPt-672034
6.9
Xgpw3109
6.7
Xgdm8
13.1
wPt-1079
49.7
2.5
2.9
1.2
.8
2.9
5.2
.9
1.4
wPt-667430
wPt-6909
wPt-2923
wPt-0485
wPt-665570
wPt-668139
wPt-733947
wPt-3480
wPt-668266
wPt-9786

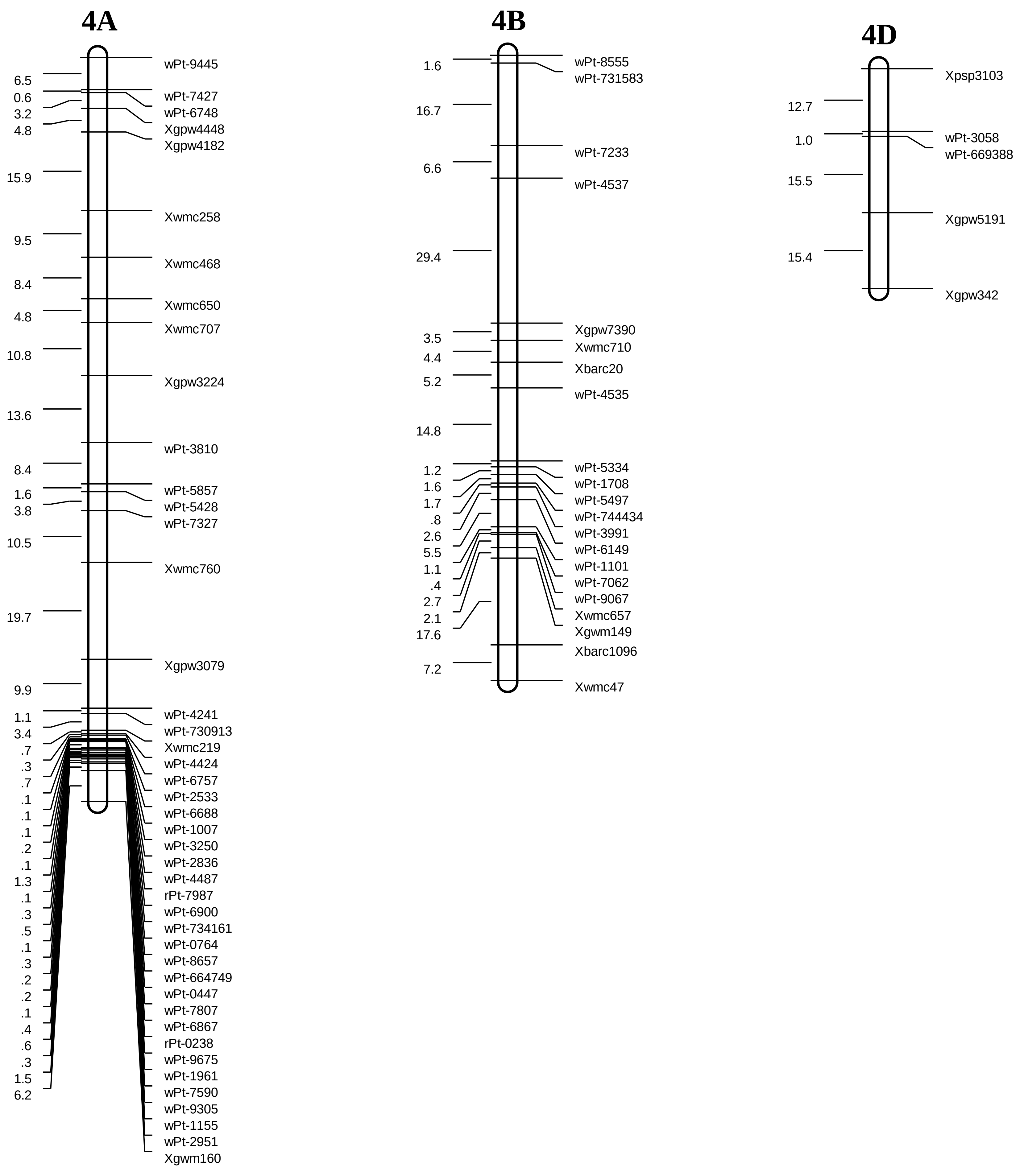
4A
6.5
0.6
3.2
4.8
15.9
9.5
8.4
4.8
10.8
13.6
8.4
1.6
3.8
10.5
19.7
9.9
1.1
3.4
.7
.3
.7
.1
.1
.1
.2
.1
1.3
.1
.3
.5
.1
.3
.2
.2
.1
.4
.6
.3
1.5
6.2
wPt-9445
wPt-7427
wPt-6748
Xgpw4448
Xgpw4182
Xwmc258
Xwmc468
Xwmc650
Xwmc707
Xgpw3224
wPt-3810
wPt-5857
wPt-5428
wPt-7327
Xwmc760
Xgpw3079
wPt-4241
wPt-730913
Xwmc219
wPt-4424
wPt-6757
wPt-2533
wPt-6688
wPt-1007
wPt-3250
wPt-2836
wPt-4487
rPt-7987
wPt-6900
wPt-734161
wPt-0764
wPt-8657
wPt-664749
wPt-0447
wPt-7807
wPt-6867
rPt-0238
wPt-9675
wPt-1961
wPt-7590
wPt-9305
wPt-1155
wPt-2951
Xgwm160
4B
1.6
16.7
6.6
29.4
3.5
4.4
5.2
14.8
1.2
1.6
1.7
.8
2.6
5.5
1.1
.4
2.7
2.1
17.6
7.2
wPt-8555
wPt-731583
wPt-7233
wPt-4537
Xgpw7390
Xwmc710
Xbarc20
wPt-4535
wPt-5334
wPt-1708
wPt-5497
wPt-744434
wPt-3991
wPt-6149
wPt-1101
wPt-7062
wPt-9067
Xwmc657
Xgwm149
Xbarc1096
Xwmc47
4D
12.7
1.0
15.5
15.4
Xpsp3103
wPt-3058
wPt-669388
Xgpw5191
Xgpw342

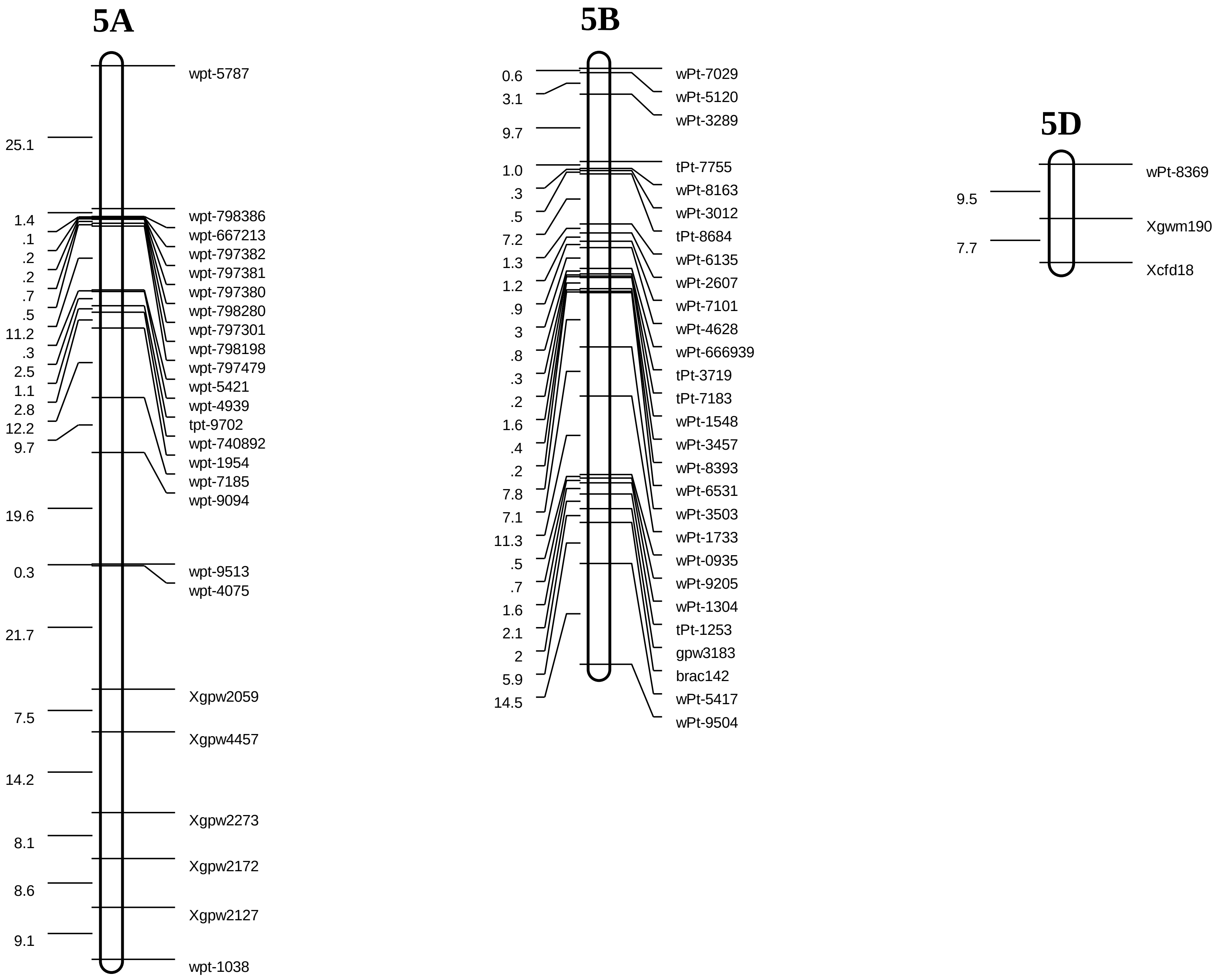
5A
wpt-5787
25.1
1.4
.1
.2
.2
.7
.5
11.2
.3
2.5
1.1
2.8
12.2
9.7
wpt-798386
wpt-667213
wpt-797382
wpt-797381
wpt-797380
wpt-798280
wpt-797301
wpt-798198
wpt-797479
wpt-5421
wpt-4939
tpt-9702
wpt-740892
wpt-1954
wpt-7185
wpt-9094
19.6
0.3
wpt-9513
wpt-4075
21.7
Xgpw2059
7.5
Xgpw4457
14.2
Xgpw2273
8.1
Xgpw2172
8.6
Xgpw2127
9.1
wpt-1038
5B
0.6
3.1
9.7
1.0
.3
.5
7.2
1.3
1.2
.9
3
.8
.3
.2
1.6
.4
.2
7.8
7.1
11.3
.5
.7
1.6
2.1
2
5.9
14.5
wPt-7029
wPt-5120
wPt-3289
tPt-7755
wPt-8163
wPt-3012
tPt-8684
wPt-6135
wPt-2607
wPt-7101
wPt-4628
wPt-666939
tPt-3719
tPt-7183
wPt-1548
wPt-3457
wPt-8393
wPt-6531
wPt-3503
wPt-1733
wPt-0935
wPt-9205
wPt-1304
tPt-1253
gpw3183
brac142
wPt-5417
wPt-9504
5D
9.5
7.7
wPt-8369
Xgwm190
Xcfd18

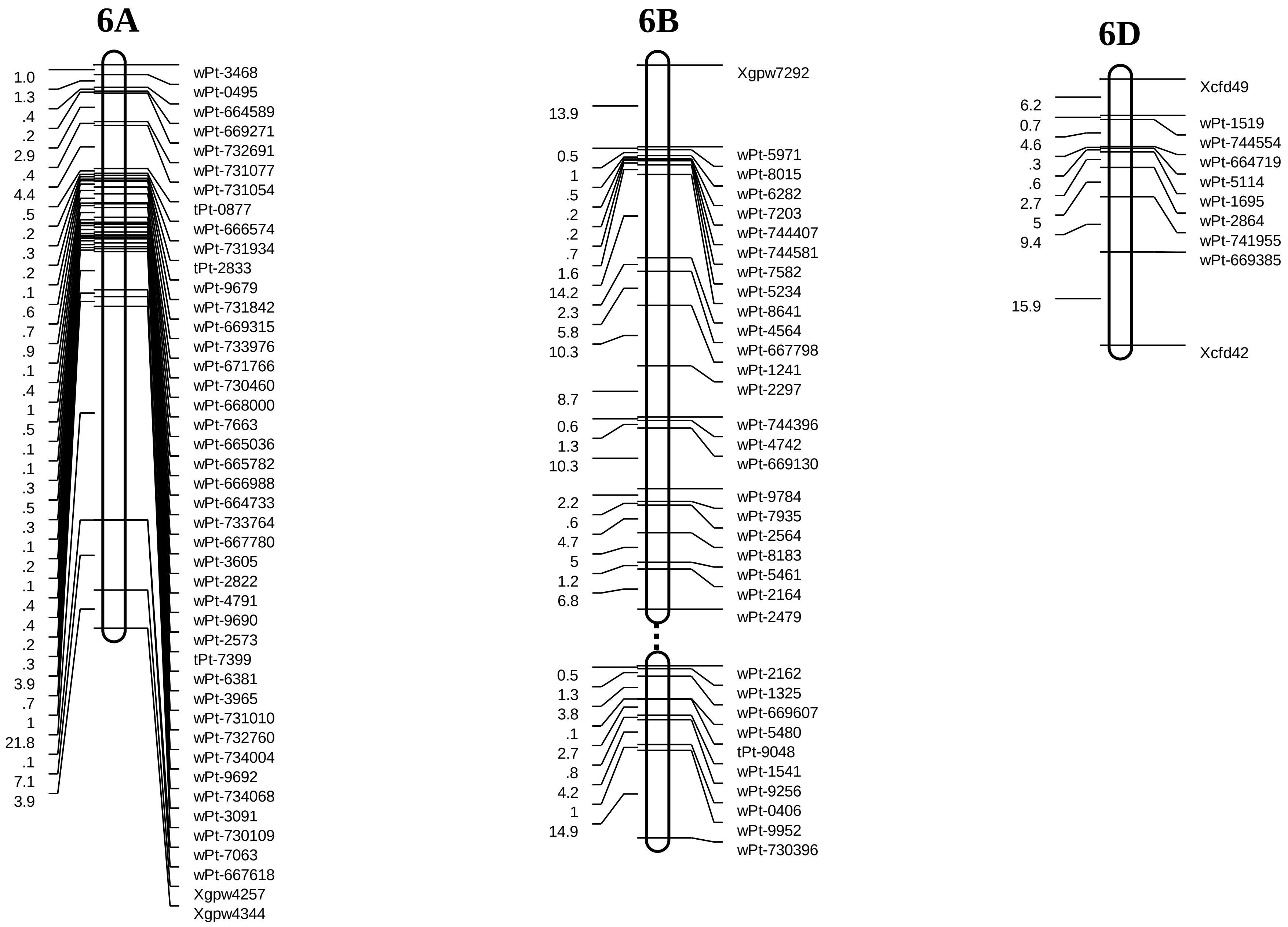

6A
1.0
1.3
.4
.2
2.9
.4
4.4
.5
.2
.3
.2
.1
.6
.7
.9
.1
.4
1
.5
.1
.1
.3
.5
.3
.1
.2
.1
.4
.4
.2
.3
3.9
.7
1
21.8
.1
7.1
3.9
wPt-3468
wPt-0495
wPt-664589
wPt-669271
wPt-732691
wPt-731077
wPt-731054
tPt-0877
wPt-666574
wPt-731934
tPt-2833
wPt-9679
wPt-731842
wPt-669315
wPt-733976
wPt-671766
wPt-730460
wPt-668000
wPt-7663
wPt-665036
wPt-665782
wPt-666988
wPt-664733
wPt-733764
wPt-667780
wPt-3605
wPt-2822
wPt-4791
wPt-9690
wPt-2573
tPt-7399
wPt-6381
wPt-3965
wPt-731010
wPt-732760
wPt-734004
wPt-9692
wPt-734068
wPt-3091
wPt-730109
wPt-7063
wPt-667618
Xgpw4257
Xgpw4344
6B
13.9
0.5
1
.5
.2
.2
.7
1.6
14.2
2.3
5.8
10.3
8.7
0.6
1.3
10.3
2.2
.6
4.7
5
1.2
6.8
0.5
1.3
3.8
.1
2.7
.8
4.2
1
14.9
Xgpw7292
wPt-5971
wPt-8015
wPt-6282
wPt-7203
wPt-744407
wPt-744581
wPt-7582
wPt-5234
wPt-8641
wPt-4564
wPt-667798
wPt-1241
wPt-2297
wPt-744396
wPt-4742
wPt-669130
wPt-9784
wPt-7935
wPt-2564
wPt-8183
wPt-5461
wPt-2164
wPt-2479
wPt-2162
wPt-1325
wPt-669607
wPt-5480
tPt-9048
wPt-1541
wPt-9256
wPt-0406
wPt-9952
wPt-730396
6D
6.2
0.7
4.6
.3
.6
2.7
5
9.4
15.9
Xcfd49
wPt-1519
wPt-744554
wPt-664719
wPt-5114
wPt-1695
wPt-2864
wPt-741955
wPt-669385
Xcfd42

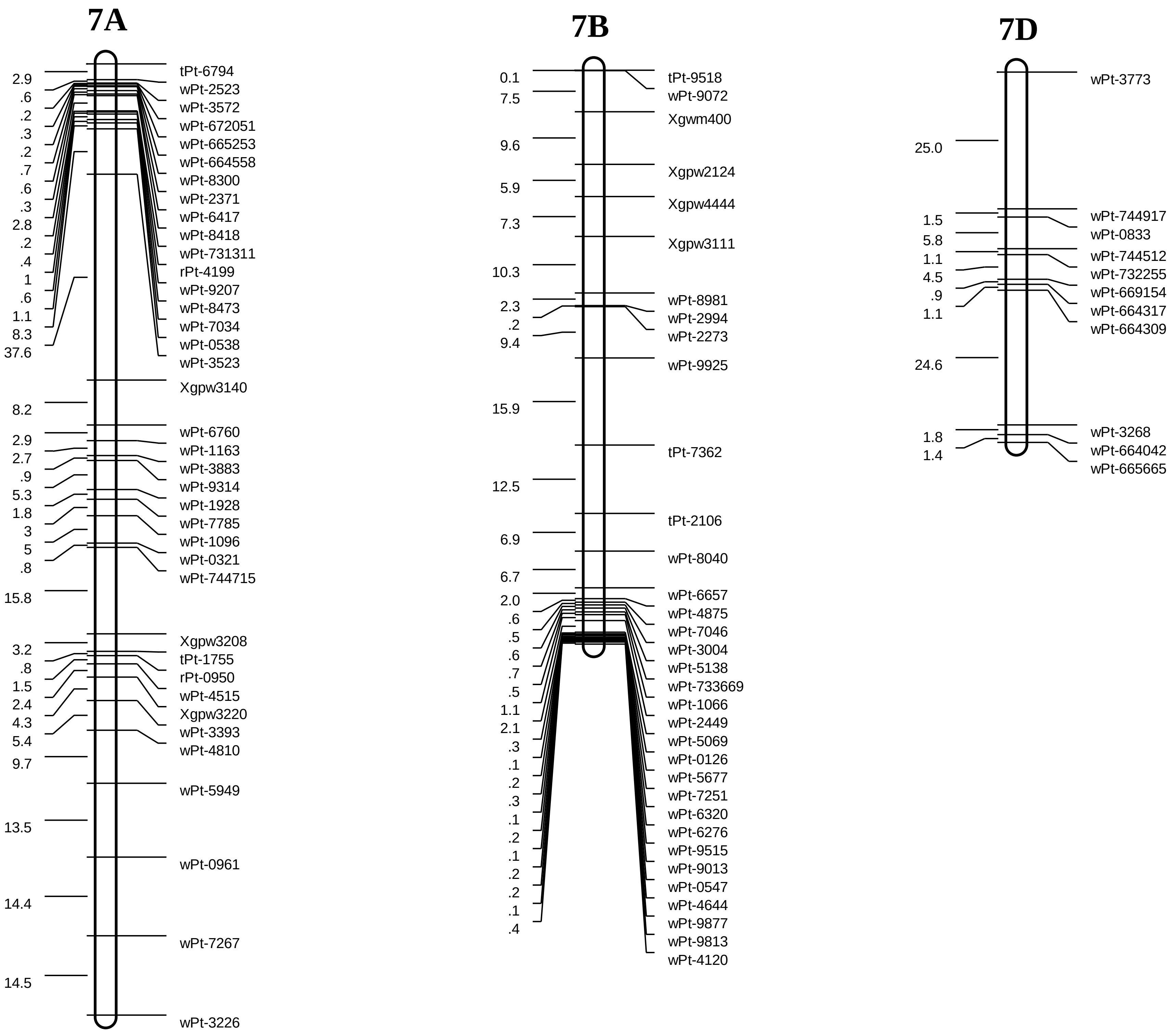

Figure S1. The linkage map of Q1028 × ZM9023 by using 564 DArT and 117 SSR makers. Numbers on the left are genetic distances in centiMorgan.
