# Supplementary material for: Genome-Wide Quantitative Trait Locus Mapping Identifies Multiple Major Loci for Brittle Rachis and Threshability in Tibetan Semi-Wild Wheat (Triticum aestivum ssp. tibetanum Shao)
Source: PLoS One. 2014 Dec 4;9(12):e114066. doi: 10.1371/journal.pone.0114066 (PMC4256410; doi:10.1371/journal.pone.0114066)
Supplement: Table S1 — The additive QTLs for brittle rachis and threshability in joint analysis of two years data by QTLNetwork 2.1. (DOCX) [file pone.0114066.s002.docx]

| Trial type | QTL | Chr.^a^ | Position^b^ | Left Marker | Right Marker | PVE (%) | Additive effect |
| --- | --- | --- | --- | --- | --- | --- | --- |
| Brittle rachis | *Qbr.sau-2D2* | 2DL | 198.3 | wPt-666518 | wPt-730613 | 18.1 | 2.5*** |
|  | *Qbr.sau-3D* | 3DS | 113.2 | wPt-9258 | Xgdm72 | 31.0 | 3.3*** |
|  | *Qbr.sau-5A* | 5AL | 114.6 | Xgpw2059 | Xgpw4457 | 17.4 | 2.1*** |
| Threshability | *Qft.sau-1B* | 1BL | 81.3 | Xgpw7383 | Xgpw2071 | 6.2 | 3.5*** |
|  | *Qft.sau-2B* | 2BS | 1.4 | wPt-6932 | wPt-4301 | 0.7 | -3.0*** |
|  | *Qft.sau-2D1* | 2DS | 74.7 | Xgpw4080 | Xgpw332 | 17.2 | 11.8*** |
|  | *Qft.sau-2D2* | 2DL | 197.3 | wPt-666518 | wPt-730613 | 12.3 | 6.1*** |
|  | *Qft.sau-5A* | 5AL | 121.1 | Xgpw4457 | Xgpw2273 | 26.9 | 13.5*** |
|  | *Qft.sau-7B* | 7B | 68.5 | tPt-7362 | tPt-2106 | 6.6 | 3.7*** |

**Table S1:** The additive QTLs for brittle rachis and threshability in joint analysis of two years data by QTLNetwork 2.1:

*** Indicates significant levels at *p* < 0.005; PVE: phenotypic variation explained.

^a^ Makers on the chromosome arm are considered to be linked with the QTL.

^b^ The position of LOD score peak in the chromosomes.
